# Membrane Lipid-Modulated Mechanism of Action and Non-Cytotoxicity of Novel Fungicide Aminoglycoside FG08

**DOI:** 10.1371/journal.pone.0073843

**Published:** 2013-09-10

**Authors:** Sanjib Shrestha, Michelle Grilley, Marina Y. Fosso, Cheng-Wei T. Chang, Jon Y. Takemoto

**Affiliations:** 1 Department of Biology, Utah State University, Logan, Utah, United States of America; 2 Department of Chemistry and Biochemistry, Logan, Utah, United States of America; 3 Synthetic Bioproducts Center (USTAR), Utah State University, North Logan, Utah, United States of America; California Department of Public Health, United States of America

## Abstract

A novel aminoglycoside, FG08, that differs from kanamycin B only by a C8 alkyl chain at the 4″-*O* position, was previously reported. Unlike kanamycin B, FG08 shows broad-spectrum fungicidal but not anti-bacterial activities. To understand its specificity for fungi, the mechanism of action of FG08 was studied using intact cells of the yeast *Saccharomyces cerevisiae* and small unilamellar membrane vesicles. With exposure to FG08 (30 µg mL^−1^), 8-fold more cells were stained with fluorescein isothiocyanate, cells had 4 to 6-fold higher K^+^ efflux rates, and 18-fold more cells were stained with SYTOX Green in comparison to exposure to kanamycin B (30 µg mL^−1^). Yeast mutants with aberrant membrane sphingolipids (no sphingoid base C4 hydroxyl group, truncated very long fatty acid chain, or lacking the terminal phosphorylinositol group of mannosyl-diinositolphosphorylphytoceramide were 4 to 8-fold less susceptible to growth inhibition with FG08 and showed 2 to 10-fold lower SYTOX Green dye uptake rates than did the isogenic wild-type strain. FG08 caused leakage of pre-loaded calcein from 50% of small unilamellar vesicles with glycerophospholipid and sterol compositions that mimic the compositions of fungal plasma membranes. Less than 5 and 10% of vesicles with glycerophospholipid and sterol compositions that mimic bacterial and mammalian cell plasma membranes, respectively, showed calcein leakage. In tetrazolium dye cytotoxicity tests, mammalian cell lines NIH3T3 and C8161.9 showed FG08 toxicity at concentrations that were 10 to 20-fold higher than fungicidal minimal inhibitory concentrations. It is concluded that FG08’s growth inhibitory specificity for fungi lie in plasma membrane permeability changes involving mechanisms that are modulated by membrane lipid composition.

## Introduction

**Figure 1:**
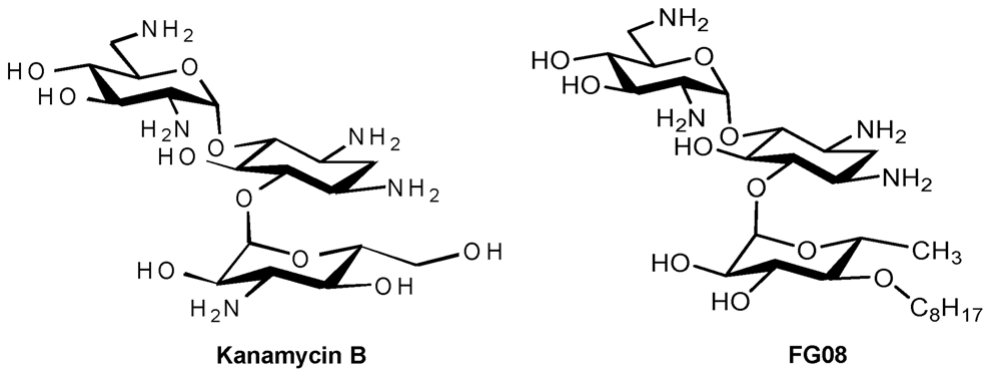
Structures of aminoglycosides kanamycin B and FG08.

Aminoglycosides are compounds having two or more amino sugars bound to an aminoacyclitol ring via glycosidic bonds. Many are used therapeutically against bacterial infections of humans and animals. Among them, kanamycin B (Figure 1), produced by the soil microbe *Streptomyces kanamyceticus*, is one of the most successful [1], [2], [3]. Kanamycin B is structurally based on sugar rings I and II of neamine with an attached ring III of *O*-6-linked kanosamine. The aminoglycosides are generally viewed to act against bacteria [3]. Most bind directly to the prokaryotic 16S rRNA in the decoding region A site, leading to the formation of defective cell proteins. Despite being mainly antibacterial, certain classical aminoglycosides are also found to inhibit crop pathogenic fungal oomycetes [4], and certain structurally unusual ones inhibit yeasts and protozoans [5], [6].

Recently, we reported a novel aminoglycoside, FG08 (Figure 1), with broad spectrum fungicidal activity [7]. FG08 differs from kanamycin B by substitution of a C8 alkyl chain at the 4″- *O* position of ring III. It, however, lacks the antibacterial properties characteristic of kanamycin B and is fungicidal to a variety of yeasts, oomycetes, and true fungi with *in vitro* minimal inhibitory concentrations (MICs) ranging between 3.9 and 31.3 µg mL^−1^ [7]. The addition, the C8 alkyl chain imparts an amphipathic character that suggests the possibility for interaction with cell membranes. Increased SYTOX Green dye uptake by FG08- treated *Fusarium graminearum* and *Candida albicans* indicate effects on the plasma membrane [7]. As a consequence, among antimicrobial aminoglycosides, FG08 appears to interact preferentially with membranes rather than ribosomes. What follows are questions about the mechanism of action on membranes and whether such a mechanism accounts for the fungicidal effects. To address these questions, it is important to determine FG08’s initial molecular target, e.g. lipid or protein or both. FG08’s structure composed of a hydroxyl and multiple amino groups suggests possible interactions with lipids or proteins with high-densities of hydrogen and ionic bonding sites exposed on the membrane external surface. Also, the molecular target(s) will need to account for FG08’s preferential inhibition of fungi over bacteria and mammalian cells.

In this study, we investigated FG08’s fungicidal mechanism of action on the plasma membrane of the yeast *S. cerevisiae*. We employed membrane-impermeable fluorescence-capable dyes and the cellular efflux of K^+^ to measure FG08 membrane pore-forming capabilities. Comparisons between wild-type and gene deletion mutants with structural or compositional defects in anionic sphingolipids were investigated to determine the influence of these lipids in FG08 action. Small unilamellar vesicles (SUVs) with glycerophospholipid and sterol compositions that mimic the lipid compositions of fungal, bacterial and mammalian cell plasma membranes were examined to determine the influence of these lipids on FG08 membrane interaction and specificity. Finally, the cell permeability and cytotoxic effects of FG08 on mammalian cells were evaluated. The results suggest that FG08’s fungicidal mechanism of action involves interaction with and perturbation of the fungal plasma membrane and that its specificity for fungal vs. mammalian and bacterial cells is influenced by membrane lipid composition.

## Materials and Methods

### FG08

FG08 was synthesized from the neamine of kanamycin B as previously described [7]. A 10 mg mL^−1^ stock solution was prepared in twice distilled water and stored at 5°C.

### Yeast Strains and Growth Medium

*S. cerevisiae* strains used in this study were W303C (MATa ade2 his3 leu2 trp1 ura3) and isogenic sphingolipid biosynthesis mutant strains W303-Δsyr2 (*MATα* ade2 his3 leu2 trp1 ura3 syr2 (sur2)::URA3), W303-Δelo2 (*MATα ade2 his3 leu2 trp1 ura3 elo2*::*HIS3*), W303-Δelo3 (MATα ade2 his3 leu2 trp1 ura3 elo2: :HIS3), W303-Δsyr4(ipt1) and (MATα ade2 his3 leu2 trp1 ura3 syr4 (ipt1)::URA3 [8], [9]. The mutants are single gene disruptants isogenic with parental strain W303C. They all have defective sphingolipids that either lack the C4-hydroxyl group of the phytosphingosine backbone (W303-Δsyr2), have truncated very long fatty acyl chains (W303-Δelo2 and W303-Δelo3), or because of the inability to add a terminal phosphoinositol group to MIPC and lack the most complex and abundant yeast sphingolipid, mannosyl-diinositolphosphorylphytoceramide (MIP_2_C) (W303-Δsyr4(ipt1). Phenotypically, these mutants lack sensitivity to the antifungal syringomycin E - a membrane lipidic pore forming cyclic lipodespsipeptide [9]. Yeast cells were grown in YPD medium (1% yeast extract, 2% peptone, and 2% glucose).

### Fluorescent Dye Staining

Yeast cells were grown for 18 h in YPD medium, and the cell density was adjusted to 1×10^7^ cells mL^−1^. Aliquots (500 µL) were taken and centrifuged for 2 min at 10,000 g. The cell pellet was suspended in 10 mM HEPES, pH 7.4, centrifuged again, and suspended in 500 µL of distilled water [7]. For fluorescein isothiocyanate (FITC) staining, FG08 was added to 100 µL of the cell suspension to give final FG08 concentrations of 30, 62.5 and 125 µg mL^−1^ (1, 2 and 4x the FG08 MIC, respectively) and incubated for 1 h at 28°C with continuous agitation. The cell suspensions were mixed with 6 µg mL^−1^ of FITC (molecular mass of 389.4, Sigma Aldrich) (10 mg mL^−1^ stock solution in acetone) for 10 min following published procedures [10]. At least 400 imaged cells were scored for fluorescence per experiment. Staining with the cationic and fluorescent nucleic acid binding dye SYTOX Green (Molecular Probes) was performed as previously described [7], [11]. SYTOX Green was added (final concentration, 0.2 µM) to 100 µL of yeast cell suspensions and allowed to stand for 10 min before addition of FG08 (30 µg mL^−1^) or kanamycin B (30 µg mL^−1^). Negative (water) and positive (Triton X-100®, 1%, vol vol^−1^) treatment controls were also prepared. Glass slides were prepared with 10 µL of each cell suspension-FITC or SYTOX Green mixture and observed in bright field and fluorescence modes using an Olympus IX81 fluorescence microscope and an Olympus MWIB filter with excitation and emission wavelengths of 488 and 512 nm, respectively. Images and data were obtained from at least two independent experiments each performed in duplicate. The percentages of stained cells were determined by comparing paired bright-field and fluorescence images.

### K^+^ efflux

Previously described K^+^ efflux measurement methods [12] were used with modification. An overnight culture of *S. cerevisiae* W303C grown in YPD medium was centrifuged at 1200 g for 5 min at room temperature. The cell pellet was washed twice with sterile deionized water. After the second wash step, the cells were suspended in sterile 10 mM HEPES-NaOH buffer, pH 6.5 with 25 mM glucose. Washed cells were used to inoculate 10 mL of sterile buffer solution to make final cell density of 1×10^7 ^CFU mL^−1^ in a sterile 50 mL capacity polyethylene tube (Corning). The cell suspension was incubated with 30 µg mL^−1^ of FG08 for 1.0, 5.0, 10, 20 and 60 min in a MaxQ 6000 incubator (ThermoFisher Scientific) at 30°C with agitation at 200 rpm. Cell-free filtrates were obtained by individually filtering each suspension with syringe filters (0.2 µm, Nalgene). The filtrates were subjected to atomic emission spectrometry using a Varian AA240 FS atomic absorption spectrometer at 766.5 nm to determine the extracellular K^+^ concentrations using a standard calibration curve based on KCl solutions. Boiled (20 min) cell suspensions were used to estimate total cellular K^+^. Untreated cells were used as negative controls. K^+^ efflux was calculated as percentage of total cellular K^+^ using the formula: K^+^ efflux (%) = ((extracellular K^+^ – extracellular K^+^ in negative control)/(total cellular K^+^))×100.

### Calcein Release from Small Unilamellar Vesicles (SUVs)

Phosphatidylcholine (PC) from *Glycine max*, L-α-phosphatidylethanolamine (PE) from *Escherichia coli*, L-α-phosphatidylglycerol (PG) (sodium salt) from egg yolk lecithin, L-α- phosphatidylinositol (PI) (sodium salt) from *G. max*, ergosterol and cholesterol were purchased from Sigma-Aldrich. SUVs were prepared by dissolving the lipids (either singly or as mixtures) in chloroform/methanol (2∶1, by vol). The mixtures were PC, PE, PI and ergosterol (5∶4:1∶2 by wt), PC and PG (7∶3 by wt), and PC and cholesterol (10∶1 by wt) that mimic the lipid compositions of fungal [11], bacterial [13], and mammalian cell plasma membranes, respectively [11]. The organic solvent was evaporated under a stream of nitrogen and the lipid mixture was allowed to dry under vacuum overnight. The dried lipid films were rehydrated in HEPES buffer (10 mM HEPES, 150 mM NaCl, pH 7.4) and 60 mM calcein (self-quenching concentration) and the suspensions were sonicated for 5 min using a bath sonicator (Sonicator™ Heat System, W-220F, Ultrasonic, Inc.) to generate SUVs with lipid concentrations at 10 mg mL^−1^ [14]. The free calcein was removed by gel filtration through a Sephadex G-50 column. FG08 at 5, 20, and 50 µg mL^−1^ were added to the calcein-loaded SUV suspensions (FG08:lipid molar ratios of 1.5∶1, 6∶1, and 14.5∶1, respectively) and calcein leakage was followed by measuring fluorescence using a Synergy HT microplate reader at an excitation wavelength of 488 nm and emission wavelength of 520 nm. Complete (100%) dye release was obtained by addition of 1% (vol vol^−1^) Triton X100®. The dye-leakage percentage was calculated as: % dye leakage = 100 (***F***−***F*** **_0_**)/(***F*** **_t_**−***F*** **_0_**), where ***F*** represents the fluorescence intensity 2 min after FG08 addition, and ***F*** **_0_** and ***F*** **_t_** represent the fluorescence intensity without FG08 and with 1% (vol vol^−1^) Triton X-100**®**, respectively [14].

### FG08 Susceptibility Testing of Wild Type and Sphingolipid Biosynthesis Mutants

Microbroth dilution assays for determination of minimal inhibitory concentrations (MICs) were conducted using the protocols of the Clinical and Laboratory Standards Institute [15]. Yeast inocula were adjusted to a final concentration of 5×10^5^ CFU mL^−1^. Drug dilutions were prepared in YPD medium broth ranging between 0.97 and 250 µg mL^−1^. For disk diffusion growth inhibitory assays, cultures were spread on YPD medium agar plate surfaces. Paper disks (0.5 cm diameter) were placed on the surfaces, and 10 µL aliquots of 1 mg mL^−1^ of FG08 solution was applied to the disks. Clear zones of inhibition were observed after incubation at 28°C for 48 h. Each test was performed in triplicate.

### Animal Cell Cytotoxicity

A C8161.9 melanoma cell line was a gift from Dr. Danny R. Welch, University of Kansas, Lawrence, KS (USA). The cells were grown in Dulbecco’s Modified Eagle Medium (DMEM)/Ham’s F12 (1∶1) containing 10% fetal bovine serum [16]. NIH3T3 cells (ATCC® CRL-1658™, American Type Culture Collection, Manassas, VA, USA.) were grown in DMEM (high glucose) media containing 10%, vol vol^−1^ fetal bovine serum in Corning Cell Bind flasks. The confluent cells were then trypsinized with 0.25% wt vol^−1^ trypsin and resuspended in fresh medium (DMEM). The cells were transferred into 96-well plates at a density of 2×10^5^ cells mL^−1^. They were mixed with FG08 at final concentrations of 10, 20, 50, 100 or 250 µg mL^−1^ or an equivalent volume of sterile double distilled water (negative control). The cells were incubated for 24 h at 37°C with 5% CO_2_ in a humidified incubator. To evaluate cell survival each well was treated with 10 µL of 3-(4,5-dimethylthiazol-2-yl)-2,5-diphenyltetrazolium bromide (MTT) (Sigma-Aldrich) for 4 h. In living cells, mitochondrial reductases convert the MTT tetrazolium to formazan which precipitates. Formazan was dissolved using acidified NaDodSO_4_ (0.01 M HCl) and quantified at A_570_ using a Synergy 4 Gen 5 spectrophotometer (BioTek). Triton X-100® (1%, vol vol^−1^) gave complete loss of cell viability and was used as the positive control. Percent cell survival was calculated as: (control value – test value) × 100/control value, where control value represents cells+MTT – drug, and test value represents cells+MTT+drug.

## Results

### Fluorescent Dye Uptake and K^+^ efflux by *S. cerevisiae* Strain W303C

**Figure 2:**
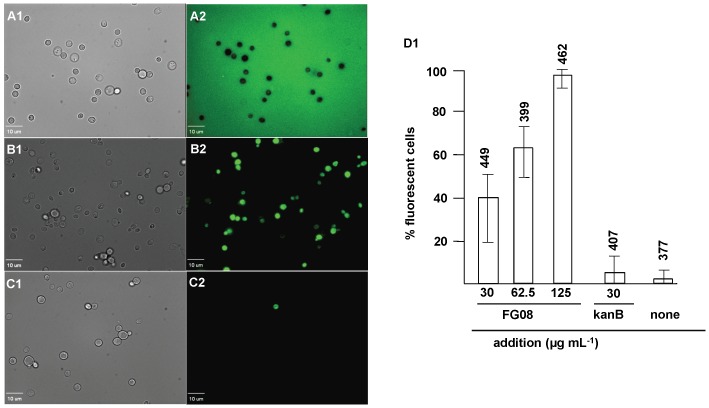
Membrane perturbation effects of FG08 on intact cells of *S. cerevisiae* strain W303C. FITC dye uptake without (A1, A2) and with FG08 (30 µg mL^− 1^) (B1, B2) or kanamycin B (30 µg mL^−1^) (C1, C2) exposure for 10 min. Bright-field images (A1, B1 and C1) are compared with fluorescence images (A2, B2, and C2) to calculate % fluorescent cells that are stained with FITC. Image A2 (no FG08 and no kanamycin B) shows no fluorescent cells against a fluorescent background. Bar length is 10 µm. Panel D1 shows dose-dependent effects of FG08 on FITC dye uptake and effects of kanamycin B and no treatment. Triton X-100® (1%, vol vol^−1^) gave 100% dye influx (data not shown). The range bars show variations in % fluorescent cells from analyses of 10 separate microscopic image fields randomly selected from at least two separate experiments. Numbers above the range bars indicate the number of cells analyzed.

With exposure to FG08 at its MIC (30 µg mL^−1^), 40% of the strain W303C cells were stained with FITC. In contrast, 8-fold fewer cells (5%) were stained when exposed to kanamycin B (30 µg mL^−1^). Unexposed cells were negligibly stained (2%) (Figure 2, 1A–C). The FG08 effect was dose-dependent with complete (100%) staining achieved with 125 µg mL^−1^ (Figure 2, D1).

**Figure 3:**
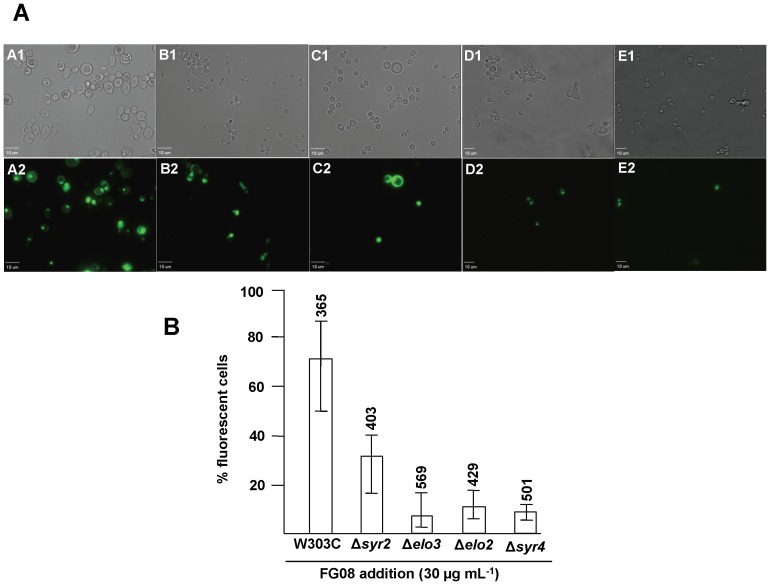
Effect of FG08 on SYTOX Green dye staining of *S. cerevisiae* strain W303C and isogenic sphingolipid biosynthetic mutants. Strain W303C (A1, A2), and isogenic mutants W303-Δ*syr2* (*sur2*) (B1, B2), W303-Δe*lo3* (C1, C2), W303-Δe*lo2* (D1, D2) and W303-Δ*syr4* (*ipt1*) (E1, E2) were exposed to FG08 (30 µg mL^−1^) for 10 min. A) Each cell observed in bright-field mode (A1–E1) was also observed for fluorescence (A2–E2). B) The % fluorescent cells in each microscopic field that stained with SYTOX Green were calculated. The range bars show variations in % fluorescent cells from analyses of 10 separate microscopic image fields randomly selected from at least two separate experiments. The numbers above the range bars indicate the total number of cells analyzed. Untreated yeast cells showed less than 3% staining. Distance bar length shows 10 µm.

**Figure 4:**
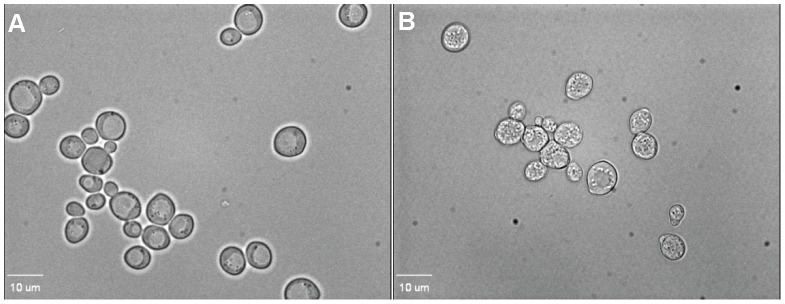
Phase contrast microscope images of FG08 effects on the cell internal structure of *S. cerevisiae* strain W303C. Cells were not treated (A) or treated (B) with FG08 (30 µg mL^−1^) for 10 min before visualization by phase contrast microscopy.

Seventy percent of the cells of strain W303C in suspension showed accumulation of SYTOX Green after a 10 min exposure to FG08 at 30 µg mL^−1^ (Figure 3, A1–A2). Six percent of the cells were stained by SYTOX Green after exposure to kanamycin B (30 µg mL^−1^) (data not shown). When observed by phase contrast microscopy and in the absence of fluorescent dye, intact cells exposed for 10 min to FG08 (30 µg mL^−1^) showed granular cytoplasmic contents not seen with untreated cells (Figure 4).

**Figure 5:**
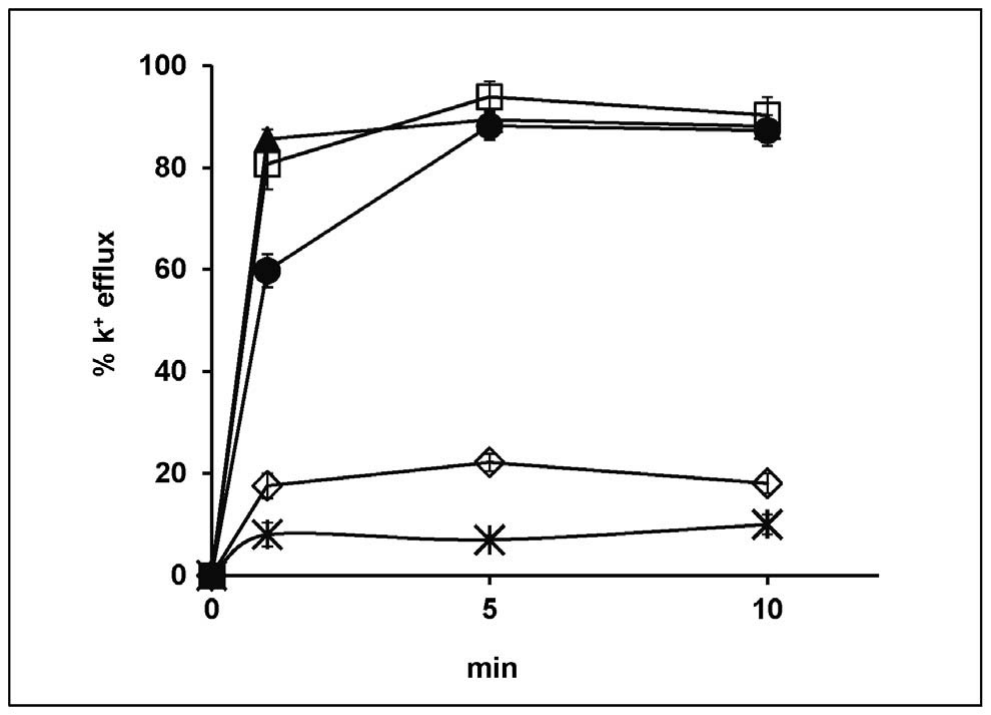
Dose-dependent effect of FG08 on cellular K^+^ efflux by *S.cerevisiae* strain W303. Cells growing in YPD medium were washed and suspended in 10-NaOH buffer, pH 6.5, 25 mM glucose with FG08 at concentrations of 20 (•), 50 (□) or 100 (▴) µg mL^−1^ or kanamycin B at 50 µg mL^−1^ (◊). Controls (×) were not treated with FG08 or kanamycin B. Error bars represent data compiled from three separate experiments.

Increased K^+^ efflux by yeast cells is observed with plasma membrane perturbation by pore forming agents [12], [13]. A rapid (half-maximum rate <1 min) efflux of K^+^ was observed when strain W303C cells were exposed to FG08 at 20 to 100 µg mL^−1^ concentrations (Figure 5). Extracellular K^+^ levels approached 90% (within 5 min) of the maximum (100%) level achieved with Triton X-100**®** (1% vol vol^−1^). Exposure to kanamycin B (50 µg mL^−1^) caused rapid efflux up to 20% of maximum level, whereas untreated cells reached 10% of maximum level.

### FG08-dependent Calcein Release by SUVs

**Figure 6:**
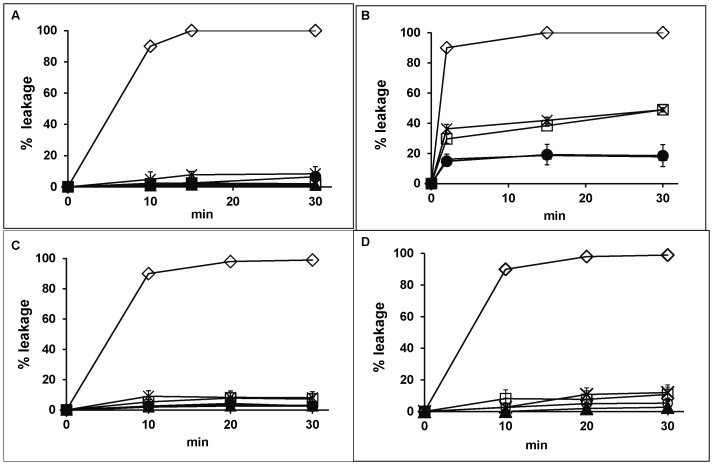
Effect of FG08 on calcein release from SUVs that mimic glyeerophospholipid and sterol compositions of fungal, bacterial, and mammalian cell plasma membranes. Calcein-loaded SUVs made with PC alone (A), or with glycerophospholipid and sterol compositions that mimic fungal (PC/PE/PI/ergosterol [5∶4:1∶2]) (B), bacterial (PC/PG [7∶3]) (C) or mammalian cell (PC/cholesterol [10∶1] (D) plasma membranes [12], [14] were exposed to FG08 at 5 (▴), 30 (□), and 62.5 (×) µg mL^−1^, kanamycin B (62.5 µg mL^−1^) (•) and Triton-X 100 (1%) (◊). Error bars represent data compiled from three separate experiments.

In order to correlate FG08’s membrane perturbation effects and its selective killing of fungi, calcein-loaded SUV model membranes representing fungal, bacterial and mammalian cell membranes were prepared and evaluated [11], [13]. Within 30 min, FG08 (30 µg mL^−1^) caused 50% calcein leakage from SUVs containing a mixture of PC, PE, PI, and ergosterol (5∶4:1∶2 by wt) (Figure 6). In contrast, at the same concentration of FG08, approximately or less than 10% leakage was observed from SUVs with PC and PG (7∶3 by wt) and with PC and cholesterol (10∶1 by wt) (Figure 6). SUV suspensions without added FG08 showed less than 2% leakage even after 2 h incubation.

### FG08 Susceptibility of Mutants with Sphingolipid Biosynthesis Defects

**Figure 7:**
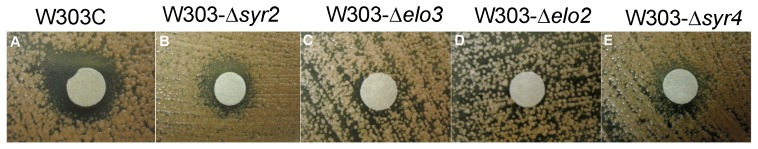
Disk-diffusion antifungal assays of FG08 against *S. cerevisiae* W303C and sphingolipid biosynthetic mutants. Ten µL aliquots of 1 mg mL^−1^ solutions of FG08 were applied to paper disks placed on YPD medium agar surfaces spread-plated with YPD medium-grown cells of *S. cerevisiae* strain W303C (A), W303-Δ*syr2 (sur2)* (B), W303-Δ*elo3* (C), W303-Δ*elo2* (D) and W303-Δ*syr4 (itp1)* (E). The plates were incubated for 48 h at 28°C.

To determine the influence of sphingolipids on FG08’s fungicidal activity, isogenic yeast mutant strains W303-Δ*syr2 (sur2)*, W303-Δ*elo3*, W303-Δ*elo2* and W303-Δ*syr4* (*ipt1*) with sphingolipid structural defects [8], [9] were examined. Strains W303-Δ*syr2 (sur2)*, W303-Δ*elo2*, W303-Δ*elo3* and W303-Δ*syr4* (*ipt1*) were all less susceptible to growth inhibition by FG08 compared to the isogenic wild-type strain W303C (Figure 7) with MICs that were 4 to 8-fold higher than for strain W303C (Table 1). With 10 min exposure to FG08 (30 µg mL^−1^), SYTOX Green stained 35%, 7%, 9.4% and 6.8% of the cells of strains W303-Δ*syr2 (sur2)*, W303-Δ*elo3*, W303-Δ*elo2*, and (W303-Δ*syr4* (*ipt1*), respectively, as compared to 70% of the strain W303C cells (Figure 3).

**Table 1:** Growth susceptibilities of yeast sphingolipid biosynthetic mutants to FG08.

Strain	MIC (µg mL^−1^)
W303C (WT)	15.6–31.3
W303-Δ*syr2(sur2)*	>125
W303-Δ*elo2*	>250
W303-Δ*elo3*	250
W303-Δ*syr4 (itp1)*	250

### Animal Cell Cytotoxicity

**Figure 8:**
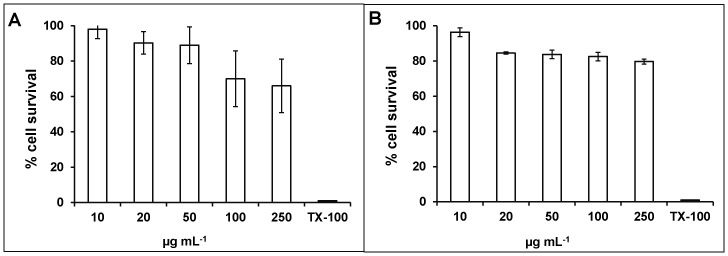
Mammalian cell cytotoxicities of FG08. MTT-based cytotoxicity assays were performed with NIH3T3 mouse fibroblast (A)) and C8161.9 melanoma (B) cells with 24 h exposure to FG08 (filled bars) at various concentrations. The positive control (100% cytotoxicity = no cell survival) was provided by treatment with Triton X-100 (1% vol vol^−1^) (open bar). Error bars represent data combined from three separate experiments.

**Figure 9:**
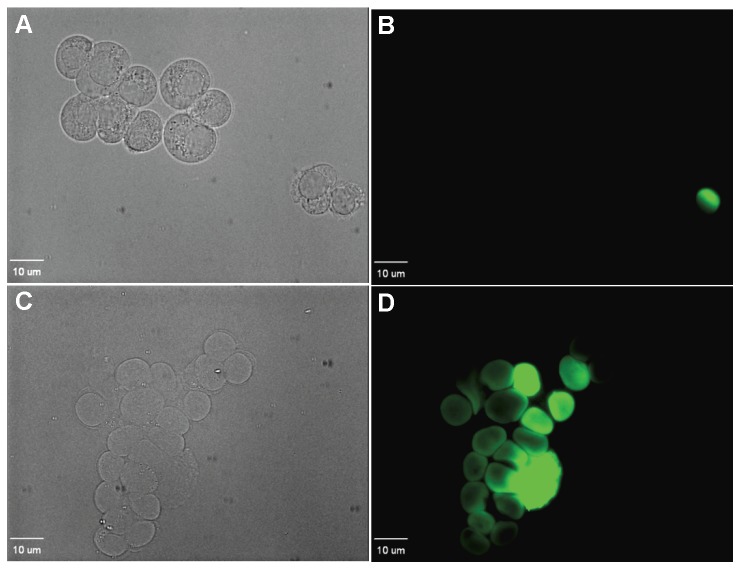
Effect of FG08 on SYTOX Green dye staining of C8161.9 cells. Cultured C6181.9 cells were exposed to 100 µg mL^−1^ FG08 and SYTOX Green and viewed microscopically in bright-field (A) and fluorescence (B) modes. Cells treated with Triton X-100® (1% vol vol^−1^) and SYTOX Green were also viewed by bright-field (C) and fluorescence (D) microscopy. Bar length is 10 µm.

FG08 concentrations needed to kill C8161.9 and NIH3T3 cells exceeded or equaled 250 µg mL^−1^, respectively (Figure 8). This was 10–20 fold higher than the antifungal MICs against wild type *S.cerevisiae* strain W303C. The membrane permeabilizing effect of FG08 was also measured on C6181.9 cells. Unlike its effect on *S. cerevisiae*, treatment of C6181.9 cells with 100 µg mL^−1^ of FG08 caused no influx of SYTOX Green dye over 30 min (Figure 9).

## Discussion

Multiple approaches were used to assess membrane-perturbation effects of FG08 on the yeast plasma membrane. The dye FITC traverses the cell surface if the plasma membrane is damaged or permeabilized by external agents and concentrates intracellularly to impart green fluorescence [10]. Similarly SYTOX Green does not cross the plasma membrane of intact yeast cells unless its permeability is compromised [7], [11]. At antifungal MICs, FG08 elicited dye uptake of both FITC and SYTOX Green by strain W303C cells within 10 min and was 12-fold more effective than kanamycin B. Similar FG08 effects on SYTOX Green dye uptake by *Fusarium graminearum* and *Candida albicans* were previously observed [7]. In addition, rapid (<5 min) and massive (∼95% of controls) effluxes of K^+^ with FG08 exposure suggest FG08 pore formation on the yeast plasma membrane. Finally, yeast cells showed granular cytoplasmic contents within 10 min of FG08 treatment with phase contrast microscopy (Figure 4). The combined observations indicate that FG08’s quick growth inhibitory effects are caused by membrane-perturbation effects that compromise normal ion and metabolite gradients across the cell surface.

To examine the influence of lipids on FG08 membrane perturbation effects and FG08’s inhibitory preference for fungi, calcein leakage studies were conducted with model membrane SUVs. SUVs were produced with glycerophospholipid and sterol compositions that mimic those of fungal (PC, PE, PI, and ergosterol), bacterial (PC and PG), and mammalian (PC and cholesterol) plasma membranes [11], [13]. FG08 caused ∼5 fold more fungal plasma membrane-mimicking SUVs to leak calcein than with bacterial and mammalian plasma membrane-mimicking SUVs. These results further indicate the membrane perturbing action of FG08. They additionally suggest an influence of lipid composition on FG08 action that in turn contributes to FG08’s growth inhibitory preference for fungi and yeasts. Compared to FG08’s MICs that inhibit yeast growth, however, about 2 times higher concentrations and longer incubation times (30 min) were needed to cause 80% leakage of calcein from fungal mimicking SUVs (Table 1 and Figure 6). These observations indicate that the studied SUV glycerophospholipid and sterol compositions influence, but by themselves do not account for all, of FG08’s yeast growth inhibitory capabilities. Other factors including other lipids (e.g. sphingolipids, see below) or membrane components likely contribute. In addition, non-membrane cell components that facilitate membrane perturbation could have roles. For example, the antifungal action of the plant defensin NaD1 involves a specific interaction with the fungal cell wall, followed by membrane permeabilization [17].

Unique phosphorylinositol-containing sphingolipids occur predominantly in the yeast plasma membrane [18]. The 3 major yeast sphingolipids are inositolphosphorylphytoceramide (IPC), mannosyl-inositolphosphorylphytoceramide (MIPC), and M(IP)_2_C with the latter two biosynthetically made by the sequential addition of mannosyl and phosphoinositol groups to IPC. All three sphingolipids possess high densities of hydrogen and ionic bonding sites for potential interaction with FG08. To determine possible influences of sphingolipids on yeast growth inhibition by FG08, biosynthetic mutants with aberrant sphingolipid structures or composition were screened against FG08. Strain W303-Δ*syr2 (sur2)* produces sphingolipids devoid of the phytosphingosine base C4 hydroxyl group. Strain W303-Δ*elo2* accumulates sphingolipids with truncated N-acylated C_22_ very long fatty acids, whereas sphingolipids of strain W303-Δ*elo3* are devoid of the C_26_ very long fatty acids. Strain W303-Δ*syr4* (*ipt1*) possesses IPC and MIPC but lacks M(IP)_2_C [8], [9] All four mutant strains were less sensitive (4 to 8-fold) to FG08 compared to the isogenic wild-type strain W303C (Figures 3 and 7) (Table 1). These differences in growth susceptibility to FG08 were further correlated with FG08 elicited cell permeability as determined by SYTOX Green uptake (Figure 7). These results indicate key roles for phosphoinositol sphingolipids in promoting FG08 action on the yeast plasma membrane. Similar roles for sphingolipids are known for the membrane pore-forming antifungal actions of *Pseudomonas syringae* lipodepsipeptide syringomycin E and plant defensin DmAMP1 [19], [20].

The influences of lipids on FG08 action could involve hydrogen and ionic bonding between FG08 and lipid head groups. FG08 has numerous hydroxyl and amino groups, and the yeast glycerophospholipid head groups and sphingolipid C4 hydroxyl and mannosyl and phosphorylinositol carbonyl and hydroxyl groups are all potential ionic and hydrogen bond donors and acceptors. While elongation of the sphingolipid very long fatty acid chain itself does not directly contribute to such interactions, it could do so indirectly by affecting the transverse position of the lipid in the membrane and therefore membrane surface accessibility of the sphingolipid head groups for bonding. Further research is needed to decipher such bonding and molecular interactions that should provide more insight into the membrane perturbation effects of FG08 and the reasons for the specific targeting of the fungal plasma membrane.

In a previous report, FG08 was not hemolytic to sheep erythrocytes even at concentrations that exceeded its antifungal MICs [7]. In the present report, we expand the descriptions of effects of FG08 on mammalian systems. It is shown that with animal cell lines NIH3T3 and C8161.9 FG08 shows low cytotoxicity and minimal membrane perturbation effects at and above FG08’s antifungal MICs. Thus, FG08 appears to be relatively non-toxic to animals as compared to fungi.

## Conclusions

FG08’s growth inhibitory specificity for fungi is suggested to lie in its ability to increase plasma membrane permeability by mechanisms that are influenced by the lipid composition of the fungal plasma membrane. FG08 has broad-spectrum antifungal activities, no antibacterial activity that would promote antibiotic resistance, and low mammalian cell toxicities. It thus appears to be a useful lead compound for the development of novel antifungal agents.
